# Chitin and Chitosan Binding to the α-Chitin Crystal: A Molecular Dynamics Study

**DOI:** 10.1021/acsomega.2c07495

**Published:** 2023-01-10

**Authors:** Magdalena Hudek, Karina Kubiak-Ossowska, Karen Johnston, Valerie A. Ferro, Paul A. Mulheran

**Affiliations:** † Department of Chemical and Process Engineering, University of Strathclyde, 75 Montrose Street, GlasgowG1 1XJ, Scotland; ‡ ARCHIE-WeSt, Department of Physics, University of Strathclyde, 107 Rottenrow East, GlasgowG4 0NG, Scotland; § Strathclyde Institute of Pharmacy and Biomedical Sciences, University of Strathclyde, 161 Cathedral Street, GlasgowG4 0RE, Scotland

## Abstract

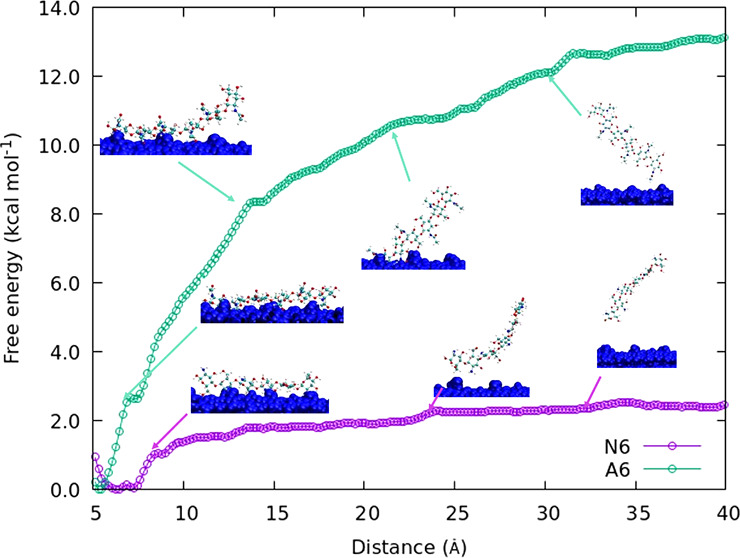

Understanding the binding of chitosan oligomers to the surface of a chitin nanocrystal is important for improving the enzymatic deacetylation of chitin and for the design of chitin/chitosan composite films. Here, we study the binding of several chito-oligomers to the (100) surface of an α-chitin crystal using molecular dynamics (MD), steered MD, and umbrella sampling. The convergence of the free energy was carefully considered and yielded a binding energies of −12.5 and −2 kcal mol^–1^ for 6-monomer-long chitin and uncharged chitosan oligomers, respectively. We also found that the results for the umbrella sampling were consistent with the force profile from the steered MD and with classical MD simulations of the adsorption process. Our results give insight into the molecular-scale interactions, which can be helpful for the design of new chitin composite films. Furthermore, the free energy curves we present can be used to validate coarse-grained models for chitin and chitosan, which are necessary to study the self-assembly of chitin crystals due to the long time scale of the process.

## Introduction

Chitin is the second most abundant polymer found in nature after cellulose. It forms the exoskeletons of arthropods and cell walls of fungi. It is also one of the most underused biomasses. It is renewable and biodegradable, leading to a variety of potential uses for chitin and its derivative chitosan, for example in wound dressings, and components of composite thin films for packaging.^1^ Using chitin as an additive in chitosan films can enhance the mechanical properties of the films. There are reports of such films with various properties and amounts of chitin added;^2^ however, theoretical studies are lacking.

Chitosan is derived from chitin via deacetylation. Current deacetylation methods use chemicals such as sodium hydroxide, which have a negative environmental impact. Furthermore, they may degrade the final product and cannot achieve full deacetylation of chitin. On the other hand, a fermentation method would be more environmentally friendly and can potentially give much greater control over the composition of the final product. As might be expected for such a common biopolymer, in nature chitinases exist that can effectively do deacetylation, but there is a need for further optimization in an industrial biotechnology setup.

**Figure 1:**
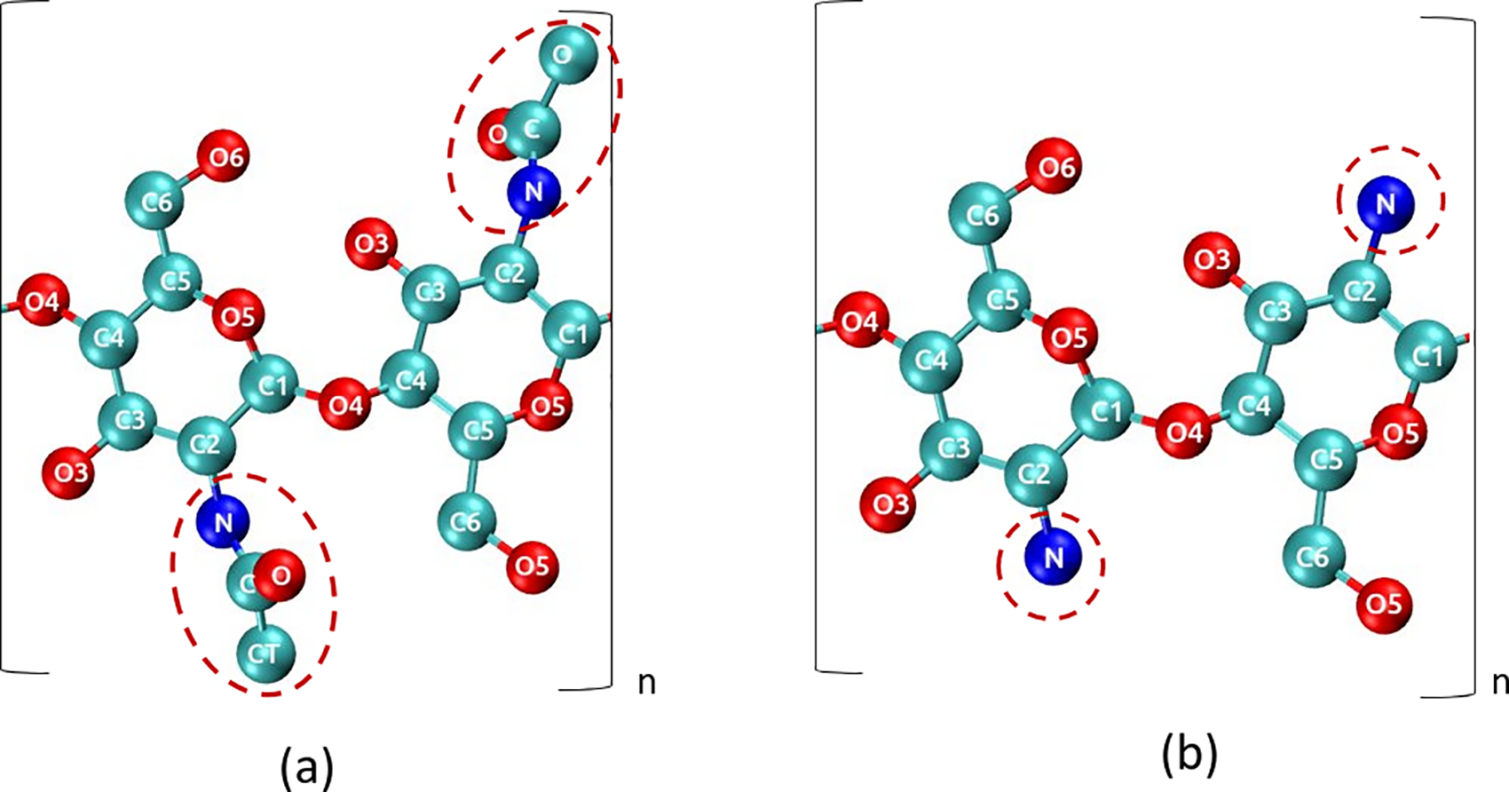
Atomic structure of chitin (a) and chitosan (b) with atom names (colored atom type C = cyan, N = blue, O = red). Hydrogens are omitted here for clarity. The naming convention used here for hydrogens adds H before its heavy atom, e.g., HO6 for hydrogen belonging to O6. The chitin chain has 2_1_ symmetry along the chain, as shown in the dimer here. The structure of chitosan (*N*-glucosamine) differs from chitin (*N*-acetylglucosamine) only in the absence of the acetyl group on the amino group, as indicated by dashed circles in red.

Chitin consists of N-β(1–4) linked acetyl-glucosamine monomers, as shown in Figure 1a. There are three chitin crystal configurations: α, β, and γ. The α-chitin crystal is the most stable configuration, where the polymer chains are stacked in an antiparallel fashion with *P*2_1_2_1_2_1_ symmetry.^3^ Intrachain hydrogen bonds stabilize the linear chitin chains: O5:HO3-O3, which has high occupancy, and O:HO-O6, which has lower occupancy^4^ (see Figure 1 for en explanation of the atom labels).

In this study, we assessed the binding of chitin and chitosan oligomers to an α-chitin nanocrystal using molecular dynamics (MD). The aim was to gain insight into the energies required to separate chitin chains from the crystal surface, which is the first step required for the digestion of chitin. Furthermore, the interaction of chitin with a chitin crystal surface provides molecular-scale understanding of the interactions involved in the production of chitin–chitosan composites.

## Methods

Molecular dynamics (MD) simulations were performed using the NAMD package^5^ (versions 2.12 and 2.15). The Charmm36^6−8^ force field was used to model the carbohydrates alongside the TIP3P water model.^9^ Visualization and postprocessing were performed in VMD^10^ and Gnuplot.^11^

### Initial Structures

The α-chitin nanocrystals as well as chitin and chitosan oligomer chains were constructed in silico, based on experimentally determined data as described below. The chitin and chitosan monomer coordinates were obtained from Naumov and Ignatov^12^ and assembled into oligomers and an α-chitin nanocrystal^13^ using an in-house Python script. The atoms names are given in Figure 1.

#### Bulk Crystal and Surface Energy in Vacuum

**Figure 2:**
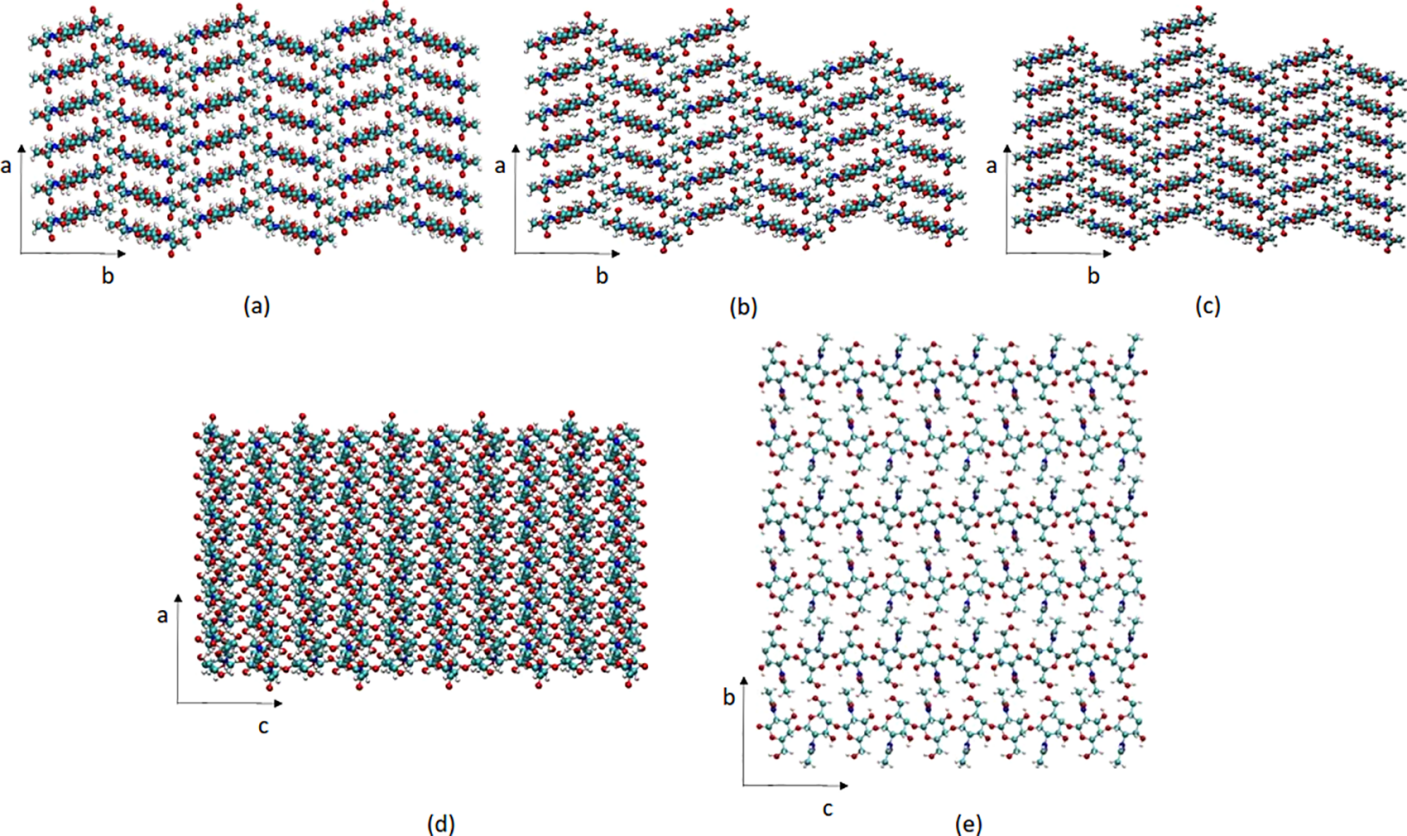
Crystal structure models used to assess the binding energy of chitin. α-Chitin crystal (a) consisting of six by six 10-monomer-long chains connected across the periodic boundary in the *c*-direction; views along other axes (d and e). This model is used to calculate the bulk crystal binding energy, and the surface energy when a vacuum gap is employed. Three chains removed from surface of crystal to obtain step configuration (b). (c) Additional chain added to the top of the surface in the antiparallel direction to assess the ideal binding energy of a single chain.

The α-chitin nanocrystal was constructed from 10-monomer-long β-1,4 linked acetylglucosamine units, arranged in a six-by-six arrangement. The chains were placed in an antiparallel fashion to obtain the desired *P*2_1_2_1_2_1_ symmetry lattice. The calculations for the binding energies of the chitin were performed in vacuum. Periodic boundary conditions (PBCs) were used in all three directions, always creating the bulk crystalline structure in the *x*,*y*-directions. A vacuum gap was added in some simulations along the *z*-axis to expose the (100) crystal surface. This surface was chosen as it presents a stable interface with water,^14^ which means that this surface is also likely to be present experimentally. Model crystals with surfaces in vacuum were constructed to study the binding energies associated with a step and an ad-chain, as can be seen in Figure 2. The step shown in Figure 2b was constructed by removing three chains from the top surface of the α-chitin crystal (Figure 2a). The ad-chain shown in Figure 2c was constructed by adding a single chain to the surface of the α-chitin crystal (Figure 2a) in the antiparallel direction to the crystal surface.

The cohesive energy per chain of the bulk crystal was calculated using the following relation: *E*_cohesive_ = *E*_36bulk_/36 – *E*_chain_, where *E*_36bulk_ is the potential energy of the bulk crystal consisting of 36 chains, and *E*_chain_ is the potential energy of a single chitin chain in a vacuum.

The total surface energy (*E*_surface_) was calculated from the difference between the potential energy of the bulk crystal (*E*_36bulk_) and that of the relaxed crystal when the vacuum gap was present. Similarly, the step energy was calculated by comparison between the potential energies of a system with 33 “bulk” chains, *E*_33_ (Figure 2b) and the surface energies of the two crystal surfaces, using the following relation: 

#### Oligomers in Solution

**Figure 3:**
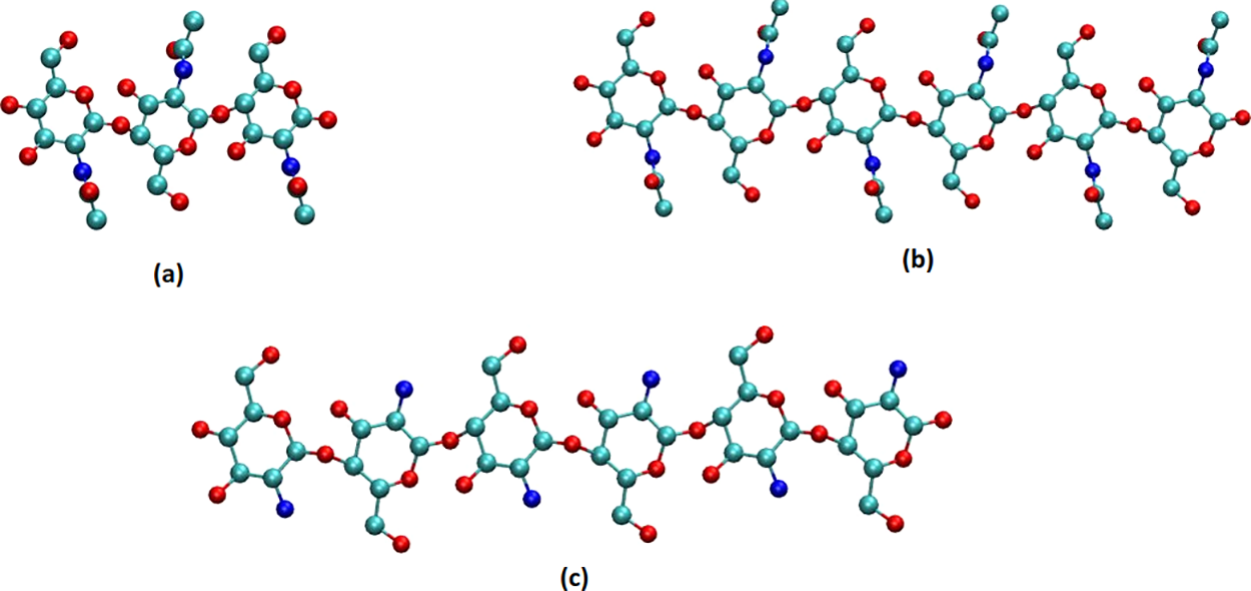
Structure of the modeled chito-oligomers: (a) A3, (b) A6, and (c) N6. A refers to the acetylglucosamine monomer of chitin and N to the neutral glucosamine monomer of chitosan.

Several chito-oligomers (oligomers consisting of acetylglucosamine and glucosamine monomers) were constructed and placed in a simulation box with the α-chitin crystal as described in the previous section. The structure of the chito-oligomers is shown in Figure 3. The oligomers were placed parallel to the (100) crystal surface to simulate the adsorbed system, and 10 Å above the crystal surface to simulate oligomers in solution. Motion of the oligomers in solution is diffusive until adsoption.

**Figure 4:**
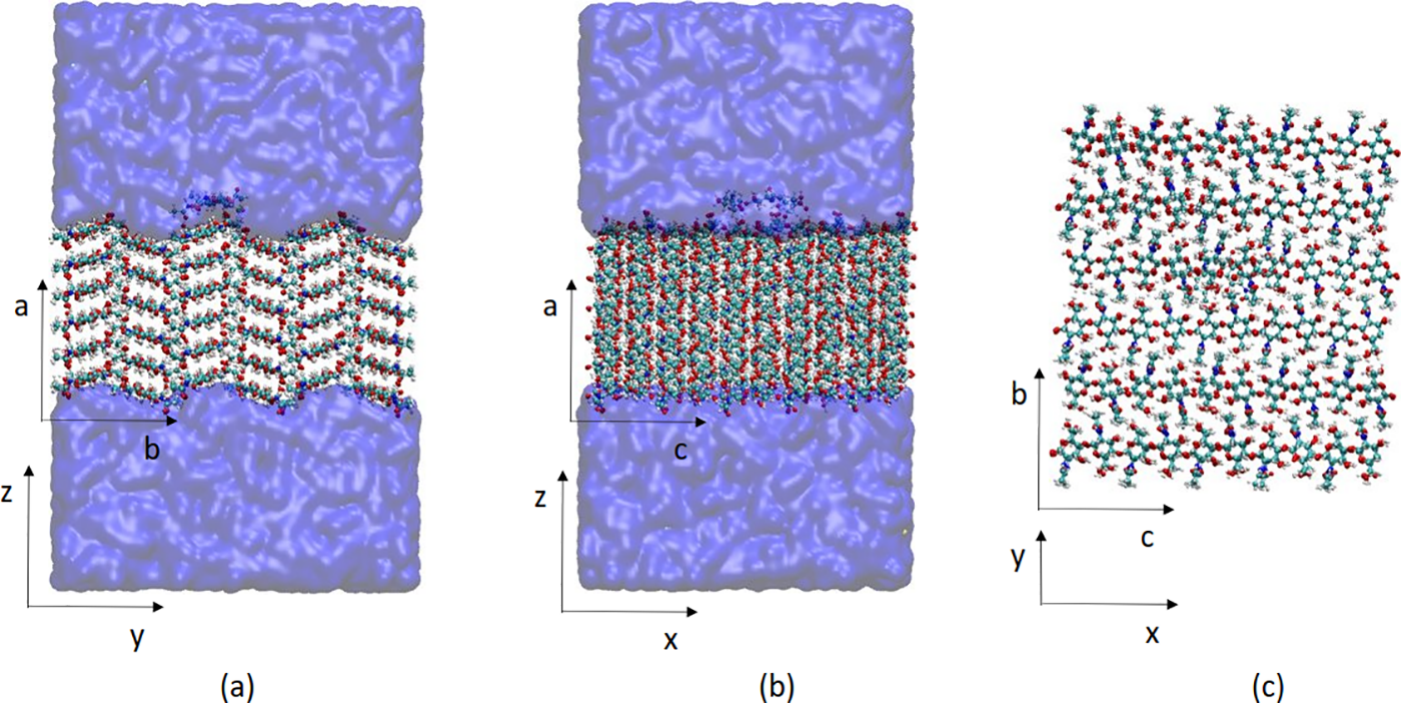
α-Chitin crystal surface with an A3 oligomer placed on the (100) crystal surface. The system is rotated such that the (100) surface is in the *x*–*y* plane in the simulations. (a) Simulation box view along the *x*-axis (*c* crystallographic axis), (b) view along the *y*-axis (*b* crystallographic axis), and (c) view from above along the *z*-axis. In panel c water is omitted for clarity.

The composite systems were solvated using TIP3P water, and 18 sodium chloride ions were added to the solution to give a concentration of 0.15 mol L^–1^. One such solvated system is shown in Figure 4.

Initially, the water underwent energy minimization for 1000 steps using the conjugate gradient algorithm, with nonsolvent atoms frozen. The water was then equilibrated for 100 ps at 300 K and 1 bar using the Langevin barostat and Langevin thermostat. Next, the entire system was minimized for a further 1000 steps and heated to 300 K over 300 ps in 10 K increments. The systems prepared in this way were then used to initiate production MD runs of various durations in the *NPT* ensemble. The Langevin thermostat with 5 ps^–1^ damping was used to control the temperature with a 1 fs time step integrator. The electrostatics were calculated using Particle Mesh Ewald with a 1.0 Å grid spacing and 12 Å cutoff for the van der Waals interactions.

### Steered Molecular Dynamics

Steered MD (SMD) simulations were performed with a pulling velocity of 1.0 Å ns^–1^ in the +*z*-direction. The C4 atom (see Figure 1) in the first monomer of the oligomer was pulled and later used as part of the collective variable for umbrella sampling. The energy (d*E*) involved in breaking a bond between the oligomer and the nanocrystal surface was calculated using
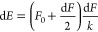
1where *F*_0_ is the force after the bond breaking, d*F* is the change in force involved with bond breaking, and the spring constant *k* = 1 kcal mol^–1^ Å^–2^.^15^

### Umbrella Sampling

Umbrella sampling was used to obtain the free energy curve (FEC) of the projection of the distance of the C4 atom along the *z*-axis. The C4 atom chosen here is the same atom used for the SMD pulling. The (100) surface of the crystal was defined as the center of mass of the C2 atoms in the top layer of the crystal. Umbrella sampling is an enhanced sampling method that enables faster phase space sampling. Due to the limitation of standard MD, we cannot always assume the ergodicity of the system, especially when a more complex energy landscape is present. For example, the system may be trapped in a metastable local energy minimum and thus cannot explore the whole phase space if the minima are several times deeper than *k*_B_*T*.^16^

Umbrella sampling is based on a series of parallel MD simulations where the system is restrained with a harmonic potential so that it can only explore a small part of phase space. Each simulation is called an umbrella or a window. The windows are evenly spaced across a reaction coordinate, which is referred to as a collective variable in the context of MD. Provided that the umbrellas overlap appropriately, this enables the system to explore all of the states along the reaction coordinate.^17^

The simulation snapshots obtained from the SMD pulling were used for the umbrella sampling set. For each system, we used 20 windows with 1 Å spacing between the windows for the A3 system and 36 windows for the A6 and N6 systems with the same spacing as before. The constants used for the harmonic potential were *k* = 2.5, 5, and 6 kcal mol^–1^ Å^–2^. The harmonic potential, *V* used for the enhanced sampling is given by *V* = (1/2)*k*(ξ – ξ_0_)^2^, where ξ is the collective variable. Here, the collective variable used is the previously described reaction coordinate.

Each window was simulated for 20 ns, with some windows extended up to 70 ns when necessary (as further explained in Results and Discussion). The FEC was then calculated using the weighted histogram method (WHAM) implementation by Grossfield.^18^

### Hydrogen Bond Analysis

The command “measure hbonds” implemented in VMD was used to calculate the number of hydrogen bonds, with a distance cutoff of 3.5 Å and 30° cutoff for the angle deviation from the 0° (180°) donor–hydrogen–acceptor angle as defined by the VMD plugin. The results were postprocessed to plot the bonds of interest and calculate their occupancy. The hydrogen bonds involving water–chitosan interactions were calculated using the VMD hydrogen bonds GUI plugin in VMD.

## Results and Discussion

### Free Energy Curves

**Figure 5:**
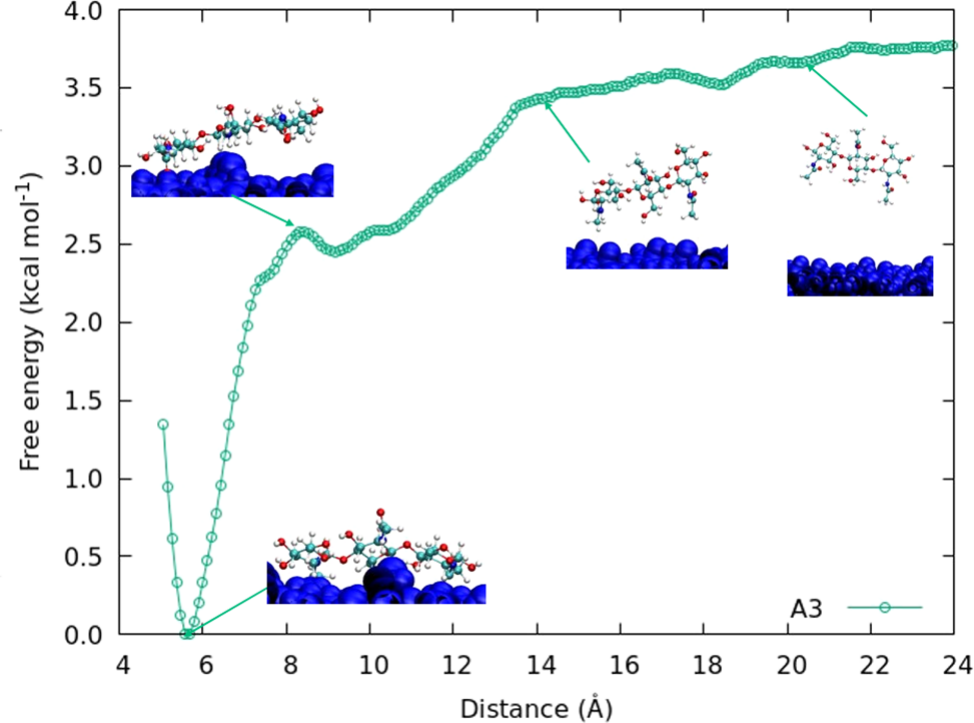
Free energy landscape for A3 oligomer, with snapshots of the system at different reaction coordinates (distances).

**Figure 6:**
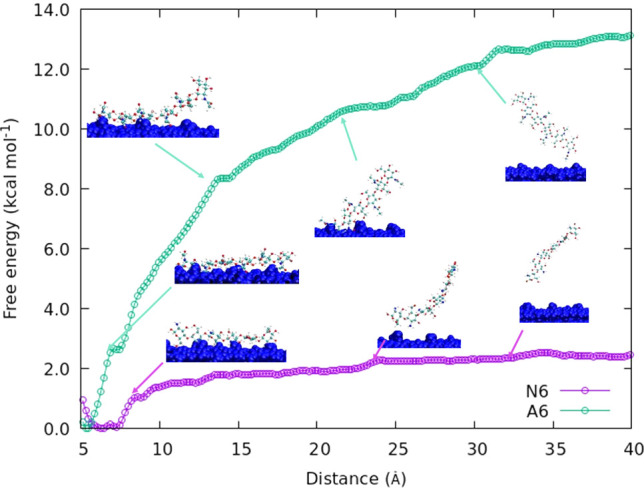
Free energy along the distance for A6 (green symbols) and N6 (purple symbols) oligomers.

The FEC obtained from the umbrella sampling for the A3 oligomer (Figure 3a) is shown in Figure 5. The FECs for the A6 and N6 oligomers (Figure 3b,c) can be seen in Figure 6. The curves calculated for chitin (A3 and A6) show good binding between the oligomer and the crystal, as indicated by the energy minima at short distances. The depth of the FEC minima is equivalent to the binding energy of the oligomer to the crystal surface. The total depth of the potential is ≈−3.8 and ≈−12.5 kcal mol^–1^ for A3 and A6, respectively. Overall the obtained FEC is relatively smooth with some minor local maxima present, which do not present significant free-energy barriers to the adsorption of the molecule from the solution. The FEC curves are approaching the asymptotic value, which is expected to be reached when no part of the oligomer is within the cutoff distance of the crystal surface. For 3-mer and 6-mers this distance is approximately 22 and 40 Å, respectively.

There is a significant difference between the FECs for A6 and N6 oligomers (Figure 6). The overall depth of the potential well of the N6 oligomer is six times lower than that of the A6 oligomer. The low binding energy between the N6 oligomer and the surface of the chitin crystal is most likely due to the absence of the acetyl group, which is not present in chitosan. Our simulations imply that acetylamino–acetylamino group binding in the *c*-crystallographic direction (intersheet in the crystal) is much stronger than the acetylamino–amino group binding, which appears to be the cause of the difference in the binding energies. The importance of the acetyl group for the oligomer–crystal binding is further explored in SMD.

The convergence of the umbrella sampling set cannot be directly assessed. Instead, we indirectly evaluate the consistency of the results. The shape of our FECs resemble the potentials of mean force (PMFs) obtained in a study of the self-assembly of cellulose nanocrystals using umbrella sampling,^19^ as can be expected for similar compounds.

The choice of reaction coordinate is one of the challenging aspects of collective variable enhanced sampling methods. The reaction coordinate must sufficiently describe the system and the studied reaction coordinate using only one dimension (or two in some instances). Here, the projection of the distance along the *z*-axis between the end C4 atom and the surface of the crystal was chosen. The benefit of this is that we mimic the spontaneously occurring adsorption process. However, a potential downside of this approach is that we cannot distinguish between adsorbed and deadsorbed states for certain ranges of the reaction coordinate.

**Figure 7:**
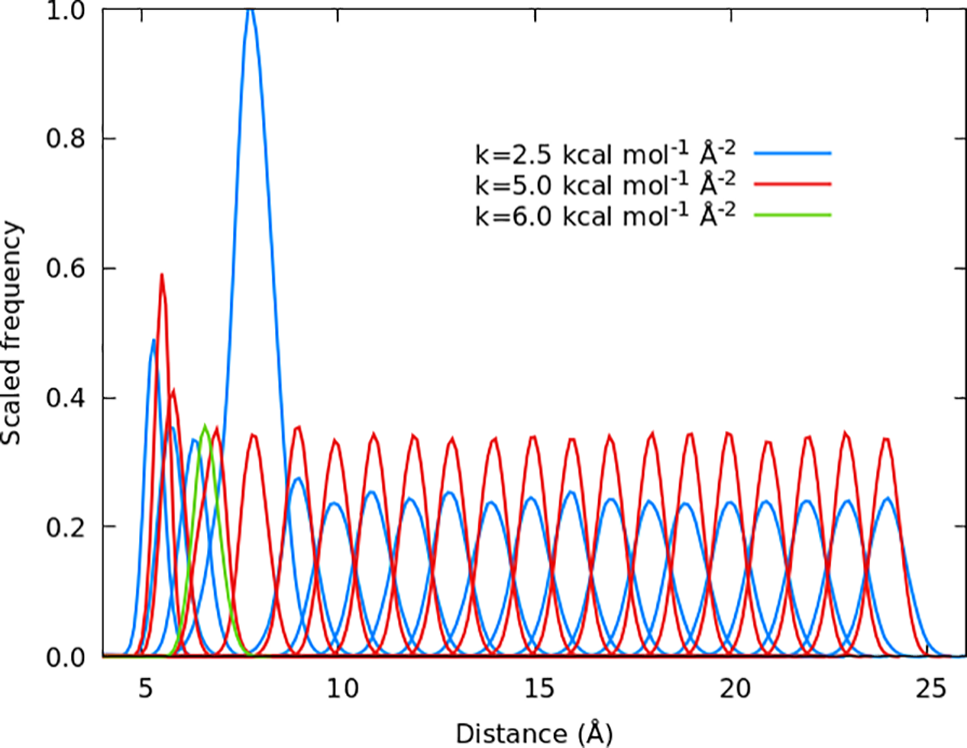
Histograms for the A3 oligomer system. Each curve on this graph is a histogram for a particular window. Blue curves correspond to the windows with *k* = 2.5 kcal mol^–1^ Å^–2^, red *k* = 5.0 kcal mol^–1^ Å^–2^, and green *k* = 6.0 kcal mol^–1^ Å^–2^.

The umbrella potential constant, *k*, and the spacing between the windows are important parameters in umbrella sampling. The umbrella sampling method relies on the sufficient overlap between the adjacent windows. The standard way to check for the window overlap along the reaction coordinate is to look at the histograms of reaction coordinate values within each window. The histograms for A3 are shown in Figure 7 and include all of the umbrella sampling windows. The histograms show very good overlap and have an approximately symmetrical appearance. The different histogram heights arise by extending the simulation within some windows as explained below. Simulations with different *k* values are also discussed below.

**Figure 8:**
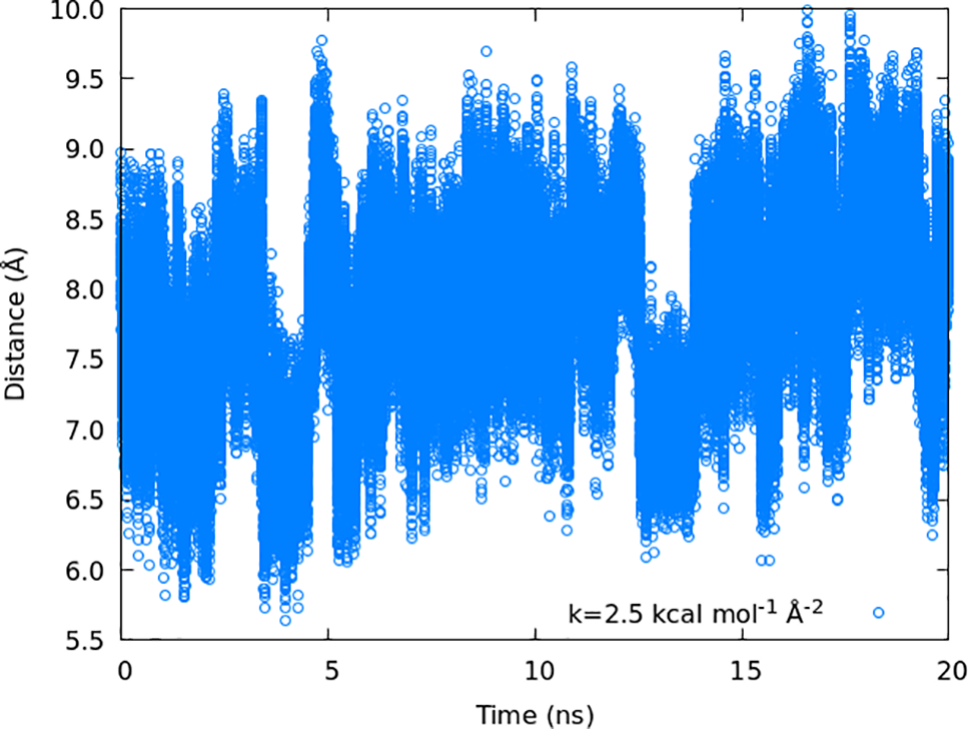
Collective variable of the window with the center 8 Å and *k* = 2.5 kcal mol^–1^ Å^–2^.

Histograms can be used to refine the choice of *k* value. An asymmetrical shape for a particular window can indicate uneven sampling of the reaction coordinate within it. In Figure 8 we look at the reaction coordinate value during the A3 simulation for the window centered at 8 Å, where a low value *k* = 2.5 kcal mol^–1^ Å^–2^ has been used. There is obvious uneven sampling in the distribution of the values of the distance with time, indicating the presence of an energy barrier (with two energetic minima at ≈7 and ≈8 Å) within the window, which the system is slow to cross at this temperature. To improve the sampling, we conducted another full set of umbrella sampling simulations with *k* = 5.0 kcal mol^–1^ Å^–2^ and additional individual windows with *k* = 6 kcal mol^–1^ Å^–2^.

**Figure 9:**
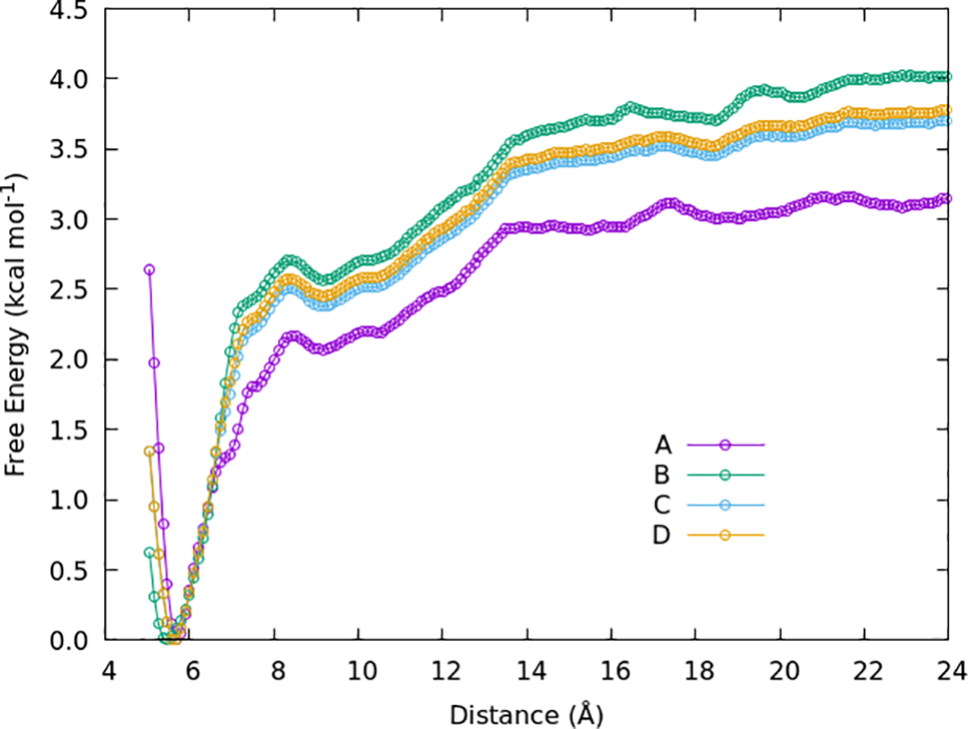
Choice of *k* value influences the final FEC. Curves A and B were obtained from the umbrella sampling sets with *k* = 5 kcal mol^–1^ Å^–2^ and *k* = 2.5 kcal mol^–1^ Å^–2^. Some windows were extended up to 70 ns per window in set B to improve phase-space sampling. Curve C was obtained by combining the two sets (A and B), while curve D was obtained the same as C, but with added window at 7 Å and *k* = 6 kcal mol^–1^ Å^–2^.

Figure 9 shows the influence of the *k* values on the final FEC. It can be seen that changing *k* within the window centered at 7 Å influences the energy values. The shape of the graph remains relatively the same, i.e., positions of the smaller energy barriers, but the height of the curve changes. The reason for this is the tendency for the system to get trapped on one side of the barrier within the window. By using a larger value of *k*, we reduce the height of this barrier, overcoming this effect. Thus, including the additional simulation with *k* = 6.0 kcal mol^–1^ Å^–2^ increases the reliability of our FEC, as we have ensured the system can now sufficiently explore the phase space along the reaction coordinate.

### Binding Energy of Chitin

In order to provide a context for the binding energy calculations, the total potential energy of some key interactions in vacuum was calculated. The cohesive energy of the chitin crystal was calculated to be −19.4 kcal mol^–1^ per monomer; this includes contributions from van der Waals (vdw) forces and electrostatics. The excess energy due to the surface was calculated to be 10.9 kcal mol^–1^ per monomer (156 mJ m^–2^). The surface energy of chitin nanocrystal^20^ determined using the contact angle method was reported to be 50 mJ m^–2^. The energy due to the step was 8.5 kcal mol^–1^ per monomer for the crystal with 33 chains, and the binding energy for the additional chain was −2.34 kcal mol^–1^ per monomer.

The binding energy of the additional chain on the top of the crystal surface supports the results we obtained from the umbrella sampling study. The short oligomer length can explain the lower depth of the energy minima in the FEC; monomers at the oligomer ends bind less effectively to the crystal surface. We expect a stronger binding for longer chains due to the higher number of hydrogen bonds and dispersion energy. The hydrogen bonds may exhibit the cooperability effect which has been observed in cellulose.^21^ This means that the strength of the hydrogen bonds is dependent on the chain length for oligomers up to chain length 7.

Strelcova et al.^4^ studied parts of chitin nanofibrils in water using MD. Chitin nanofibrils are naturally assembled chitin chains usually consisting of 18–25 chitin chains approximately 570 monomers in length. They calculated the total contribution to binding per single monomer to be −8.7 kcal mol^–1^ using MM/PBSA postprocessing method implemented in Amber for 20-monomer-long chains. This value is expected to differ from ours as the chitin fibrils differ in structure from the α-chitin crystal due to their smaller and finite size. The nanofibrils typically have a polygonal surface with a large surface area of the crystal exposed to the solvent. Nevertheless, this shows that our results are in line with these other calculations, given the inherent approximations of the potential model employed herein.

### MD

It is expected that chito-oligomers will adsorb to the chitin crystal surface at a pH level higher than 6, at which they are mainly neutral. In our unbiased MD simulations, the neutral oligomers adsorbed on the surface and did not spontaneously desorb into the solution. We also explored the behavior of a charged chitosan oligomer, which we obtained by protonating the amino groups of the N6 oligomer. This classical MD simulation was performed to verify that the chitosan oligomer we constructed would desorb from the crystal into the solution at a pH value below 6; indeed the charged chitosan oligomer desorbs from the chitin crystal surface. The other oligomers showed different mobility on the crystal surface, but remained adsorbed for the duration of the simulations.

### SMD

**Figure 10:**
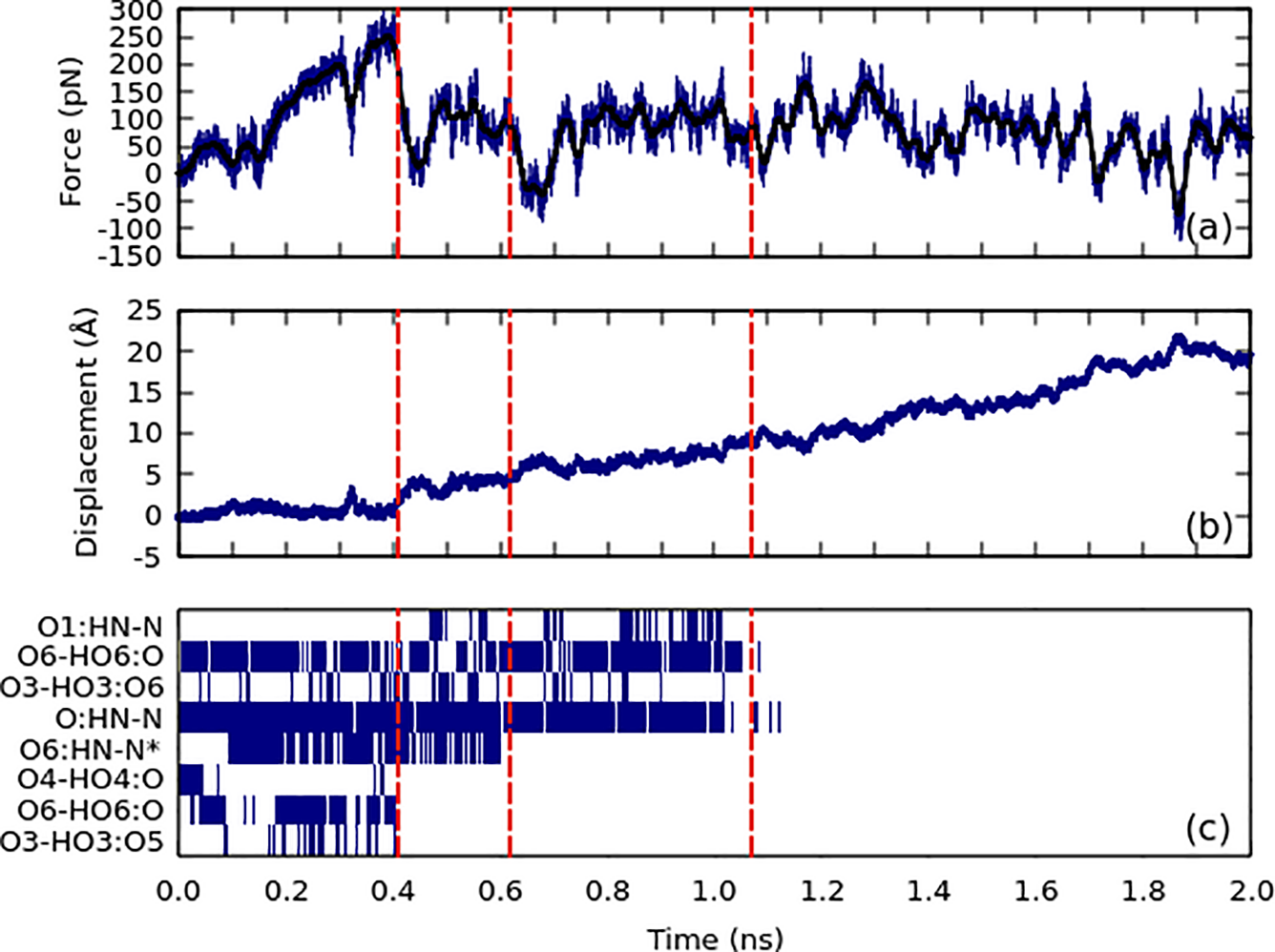
Force–time (a) and displacement–time (b) graphs for the A3 oligomer pulled from the surface of the crystal with constant velocity. (c) Occupancy of hydrogen bonds during the simulation. The times of interest are marked with red vertical lines.

In constant velocity SMD, the spring force varies during the simulation, as shown in Figure 10a for the A3 oligomer SMD. We can correlate the drops in force and sharp increases in the spring extension (Figure 10b) with the breaking of certain hydrogen bonds and with the change in the glycosidic bond conformation as seen in Table 1. The intrachain hydrogen bonds arise as the consequence of the steric effects (exoanomeric effect) and stabilize the 2_1_ chain configuration. After the breaking of the hydrogen bonds between the crystal and the oligomer, conformational changes of the oligomer occur. This explains the changes in force observed after the oligomer has been completely pulled from the crystal.

**Table 1:** SMD A3 and A6 Energies and Hydrogen Bonds Broken during the Simulation, Where * Indicates Intrachain Hydrogen Bond

Time (ns)	d*E*(kcal mol^–1^)	H-bonds
A3		
0.41	6.5	O3-HO3:O5, O6-HO6:O, O4-HO4:O
0.62	0.7	O6-HN-N*
1.10	1.7	O6-HO6:O
A6		
0.45	3.0	O:HN1-N
1.10	4.9	O:HN1-N × 2
1.50	2.2	O:HN1-N (O6:HO6-O6)
2.50	2.2	O:HN1-N × 2

The O6-HO6-O and O:HN-N bonds contributed more significantly toward the oligomer–crystal binding than the rest of the hydrogen bonds, as seen from their high occupancies prior to their breaking in Figure 10c. Interestingly, one bond-breaking event observed during the SMD pulling at 0.62 ns was breaking the interchain hydrogen bond O6-HO6:N*, where * refers to the intrachain hydrogen bond.

The hydrogen bond analysis also suggests that the intrachain hydrogen bonds are mostly preserved during the pulling, indicating that the oligomer’s 2-fold linear structure is mostly conserved. Similar results were observed for the rest of the A6 oligomer as shown in Table. 1.

### Readsorption

Once pulled clear from the surface using SMD, the A3 and A6 oligomers were released, and unbiased MD simulations were performed to see if the oligomers spontaneously readsorb. Despite the shape of the free energy landscapes in Figure 5 and Figure 6, this adsorption is expected to be slow (on the MD time scale of 100 ns) due to kinetic effects and the orientational requirements for successful adsorption. We observed adsorption events after 65 and 70 ns for the A3 and A6 oligomers, respectively.

The A6 simulation was extended to 100 ns, during which the oligomer remained adsorbed. This indicates strong adsorption, where the oligomer remains adsorbed without external forces introduced in the system, which is consistent with our umbrella sampling results.

The potential energy of the simulation was monitored to see how it changes upon the oligomer adsorption, to again obtain an indication of the magnitude of the adsorption free energy.^22^ The potential energy values rapidly oscillate around their mean value. The time average of the different parts of the simulations was calculated to evaluate the change in the potential energy of the whole system due to oligomer adsorption. The difference in potential energy in the part of the simulation where the A6 oligomer is adsorbed and the part where it is free in solution is −14.2 ± 1.3 or −2.4 ± 0.2 kcal mol^–1^ per monomer, consistent with the adsorption energy values of −12.2 kcal mol^–1^. The time-averaged potential energy difference for A3 oligomer is −9.8 ± 1.3 or −3.3 ± 0.4 kcal mol^–1^ per monomer. Again this adsorption value supports that from the FEC calculations.

The A6 oligomer is adsorbed in the *b*-direction across the crystal surface, which is not the most energetically favorable. This finding is in agreement with a study by Yudin et al.,^2^ who used MD alongside experimental methods to assess the orientation of a chitosan chain on the chitin crystal surface. Although the details of the MD methodology were unclear, the experimental and theoretical results indicated the strongest binding when the chitosan chain had a parallel or antiparallel orientation along the crystal surface, lining up with the chains on the crystal. This parallel or antiparallel orientation (in the *c*- direction) enables the formation of the highest number of hydrogen bonds. We see such adsorption for the A3 oligomer. The total number of hydrogen bonds in the system has been calculated. For A6 and A3 there are on average 11.7 ± 0.5 and 1.2 ± 0.3 fewer bonds, respectively, in the adsorbed state than in the desorbed state.

## Conclusion

We used MD simulations to study the binding energy of chito-oligomers with a model α-chitin surface. The free energy landscapes of the chitin trimer, and chitin and chitosan hexamers adsorbing to the crystal surface using umbrella sampling were calculated. The oligomer–crystal binding energies were −12.5 and −2 kcal mol^–1^ for chitin and chitosan 6-monomer-long oligomer chains, respectively. To validate free energy calculations, several additional calculations were performed. First, the binding energy of chitin crystal and excess surface energy in a vacuum were calculated. Then, using SMD pulling, the strength of the chitin oligomer binding to the chitin crystal surface and the breaking of the relevant hydrogen bonds was evaluated. The SMD pulling disrupted the intrachain bonding and caused conformational changes in the oligomer. This explains the slow adsorption process observed during classic MD simulations. Our results align with similar studies carried out for cellulose and chitin nanofibrils.^4,19^

The calculations performed here help shed light on the dynamics of chito-oligomers and their adsorption to the chitin crystal. The slow dynamics of the crystal–oligomer interactions mean that it is challenging to study the self-assembly of chitin nanofibrils and crystals using classical MD. Our FECs can be used to further the understanding of these processes. The FECs can also be helpful when constructing and validating coarse-grained models for chitin and chitosan, which can span into the nanofibril length scale and microsecond time scale. Furthermore, understanding the material properties at the nanoscale is very valuable when designing novel materials and processes, such as composite chitin thin films and enzymes engineered for the production of chitosan.
